# Kaempferol: A potential agent in the prevention of colorectal cancer

**DOI:** 10.14814/phy2.15488

**Published:** 2022-10-18

**Authors:** Hamid Reza Nejabati, Leila Roshangar

**Affiliations:** ^1^ Stem Cell Research Center Tabriz University of Medical Sciences Tabriz Iran

**Keywords:** anticancer, colorectal cancer, flavonol, kaempferol

## Abstract

Colorectal cancer (CRC) is the third most prevalent cancer in relation to incidence and mortality rate and its incidence is considerably increasing annually due to the change in the dietary habit and lifestyle of the world population. Although conventional therapeutic options, such as surgery, chemo‐ and radiotherapy have profound impacts on the treatment of CRC, dietary therapeutic agents, particularly natural products have been regarded as the safest alternatives for the treatment of CRC. Kaempferol (KMP), a naturally derived flavonol, has been shown to reduce the production of reactive oxygen species (ROS), such as superoxide ions, hydroxyl radicals, and reactive nitrogen species (RNS), especially peroxynitrite. Furthermore, this flavonol inhibits xanthine oxidase (XO) activity and increases the activities of catalase, heme oxygenase‐1 (HO), and superoxide dismutase (SOD) in a wide range of cancer and non‐cancer cells. Based on several studies, KMP is also a hopeful anticancer which carries out its anticancer action via suppression of angiogenesis, stimulation of apoptosis, and cell cycle arrest. Due to various applications of KMP as an anticancer flavonol, this review article aims to highlight the current knowledge regarding the role of KMP in CRC.

## Graphical abstract

Overall, a good deal of preclinical studies has approved the function of KMP in the treatment of CRC. However, there are still numerous doubts regarding the therapeutic potency of KMP in CRC therapy which should be addressed by elite scientists all over the world.

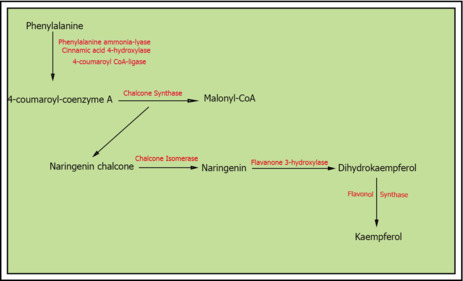

## 1 INTRODUCTION

Over the past few years, cancer research has made great strides toward discovering novel therapeutic targets; however, nowadays, cancer is classified as one of the most life‐threatening disease diseases at a global scale and there is a long road ahead of us for entirely defeating it. In addition to the conventional therapeutic options, particularly chemo‐ and radiotherapy, a number of newly established alternatives, including immunotherapy, targeted therapy using nanomedicine, and targeting noncoding RNAs (ncRNAs) (Madni et al., 2017; Mollaei et al., 2019; Yang, 2015). In this regard, natural products, especially flavonoids have been extensively studied on different kinds of cancer in recent years (Giordano & Tommonaro, 2019).

Colorectal cancer (CRC) is the third most prevalent cancer in relation to incidence and mortality rate (Bray et al., 2018) and its incidence is considerably increasing annually due to the change in the dietary habit and lifestyle of the world population (Aquina et al., 2017). In general, CRC is categorized into three classes, including sporadic, inflammation‐dependent, and familial CRC based on their molecular mechanisms of tumor formation and progression (Narayan & Roy, 2003; Roncucci & Mariani, 2015) among which sporadic CRC has the highest prevalence (nearly 75 percent) (Yamagishi et al., 2016). Chronic inflammation contributes greatly to tumorigenesis of the inflammation‐dependent CRC which occurs in patients with inflammatory bowel disease (IBD) (Grivennikov & Cominelli, 2016; Issa & Noureddine, 2017). Although conventional therapeutic options, such as surgery, chemo‐ and radiotherapy have profound impacts on the treatment of CRC, drug resistance and toxicities remain as immense challenges. Therefore, dietary therapeutic agents, particularly natural products may have been regarded as the safest alternatives for the treatment of CRC, which could ameliorate the indications and life quality of patients with CRC (Akiyama et al., 2018; Bar‐Shalom et al., 2019).

It is increasingly evident that dietary intake of fruit and vegetables, because of their bioactive compounds, has numerous beneficial health effects and has an active role in the prevention of pathological conditions, in particular cancer, diabetes, allergy, and cardiovascular disease (Ghosh et al., 2014; Majeed et al., 2021). Flavonoids are one of these most important compounds among polyphenolic secondary metabolites and nutrients which are further categorized into six subclasses as a result of their some extra modifications, including glycosylation and hydroxylation (Dai & Mumper, 2010; D'archivio et al., 2007). Flavonols, the most popular flavonoids in plant food, are the main subgroup of flavonoids that have appealing biological roles, especially their antioxidative, antibacterial, anti‐inflammatory, and anticancer functions (Bangar et al., 2022).

Kaempferol (KMP), a naturally derived flavonol, reduces the production of Reactive Oxygen Species (ROS), such as superoxide ions, hydroxyl radical, and Reactive Nitrogen Species (RNS), especially peroxynitrite (Wang et al., 2006). It is highlighted that the activities of xanthine oxidase (XO) (Li et al., 2021), heme oxygenase‐1 (HO) (Gamage et al., 2022), and superoxide dismutase (SOD) are highly associated with the development and progression of CRC. KMP has been shown to inhibit XO activity and increases the activities of catalase, HO, and SOD (Heijnen et al., 2001; Klaunig & Kamendulis, 2004) in a wide range of cancer and non‐cancer cells. While the promoting effects of KMP on the levels of SOD in a rat model of CRC were reported (Nirmala & Ramanathan, 2011), it has yet to be known the exact effects of KMP on the activities of HO‐1 and XO in CRC. Based on several studies, KMP is a hopeful anticancer agent (Alam et al., 2020; Rajendran et al., 2014) which carries out its anticancer action via suppression of angiogenesis, stimulation of apoptosis, and cell cycle arrest (Huang et al., 2013; Kang et al., 2010). Due to the various application of KMP as an anticancer flavonol, this review article aims to highlight the current knowledge regarding the role of KMP in CRC.

## 2 KMP

### 2.1 Chemistry of KMP

KMP (3,4′,5,7‐tetrahydroxyflavone), also well‐known as indigo yellow, is a nutritional compound that is amply present in a broad range of plants, including Delphinium, Camellia, berberis, Citrus, Equisetum spp, Sophora japonica, and fruits or vegetables, such as teas, cabbage, broccoli, apples, grapes, citrus fruits, strawberries, beans, tomatoes, and onions (Bangar et al., 2022; Imran, Rauf, et al., 2019; Imran, Salehi, et al., 2019). The presence of phenolic compounds in the chemical structure of the above‐mentioned nutrients has been connected to their therapeutic capacities, especially their antioxidant activity (Bangar et al., 2022). The presence of a connection between molecular structure and pharmacological activity always manifests noteworthy information. KMP has been the subject of various empirical and theoretical research among several compounds (Bangar et al., 2022). The biological activities of polyphenols stem largely from their molecular shape and relative spatial orientation of phenolic rings and hydroxyl groups. Therefore, the exact identification of molecular geometry is immensely essential for clarifying the mechanism underlying the actions of natural antioxidants (Bangar et al., 2022).

### 2.2 Biosynthesis of KMP

**FIGURE 1:**
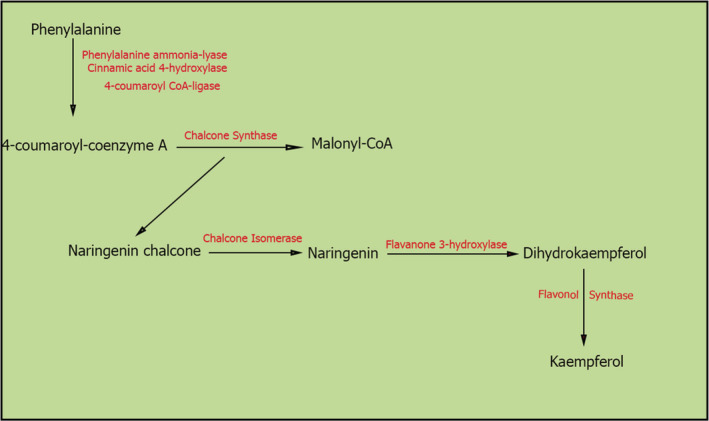
Biosynthetic pathway of Kaempferol.

KMP, which presents as diphenyl propane conformation (C6‐C3‐C6), is generated with the assistance of multiple enzymes (Bangar et al., 2022) (Figure 1). Firstly, phenylalanine is converted to 4‐coumaroyl‐coenzyme A (CoA) via the participation of phenylalanine ammonia‐lyase (PAL), cinnamic acid 4‐hydroxylase (C4H1), and 4‐coumaroyl CoA‐ligase (4CL). The result of the reaction between three molecules of malonyl CoA acquired from the first metabolism plus one molecule of 4‐coumaroryl‐CoA, catalyzed by chalcone synthase (CHS), is the production of a flavonoid naringenin chalcone (Chen et al., 2021), which is then isomerized into naringenin via chalcone isomerase (CHI) (Jones et al., 2016; Xiong et al., 2017). The addition of a hydroxyl group at the C3 position in naringenin through flavanone 3‐hydroxylase (F3H), results in the formation of dihydrokaempferol. In the last phase, the introduction of a double bond at the C2‐C3 position generates KMP via the action of flavonol synthase (FLS) (Duan et al., 2017; Malla et al., 2013). Sugar fractions, including glucose, galactose, and rhamnose are commonly bound to KMP to form glycosides, one of which is kaempferol‐3‐O‐glucoside, named astragalin (Pei et al., 2016).

### 2.3 Metabolism and absorption of KMP

KMP shows lipophilic properties when existing in free form, while the glycosylated KMP exhibits lipophobic characteristics due to its attaching to sugar portions (Jiang et al., 2015). The presence of hydroxyl groups in the structure of KMP makes it more likely to generate bonds with sugar fractions, titled O‐glycosides. The most common sugar molecules, which are generally related to KMP, are glucose, galactose, arabinose, xylose, rhamnose, and rutinoside (Xiao et al., 2014).

The lipid solubility of flavonols has a critical role in its bioavailability. Because of the lipophilic character of KMP, the intestinal absorption of KMP can take place via passive or active transport (Crespy et al., 2003). Both in vitro and in vivo studies have demonstrated that the absorbability of KMP glycosides is considerably poorer than those aglycones (Lehtonen et al., 2010). After intake of a KMP‐rich diet, its aglycones and glycoside form are absorbed in various ways. Lipophilic aglycones travel unmetabolized via the gut and penetrate the enterocytes, from where they may either be metabolized before absorption or they could be immediately absorbed (Williamson et al., 2018). Aglycones metabolism occurs in two phases; the first one requires two procedures, in particular oxidation, and O‐demethylation. The second one comprises sulfation, glucuronidation, and methylation, which are mediated through multiple enzymes, such as sulfotransferases, uridine‐5‐diphosphate glucuronosyltransferases, and catechol‐O methyltransferases (Alvarez et al., 2010).

Lipophobic KMP glycosides should be turned into aglycone form, which is mediated by Lactase‐phlorizin hydrolase (LPH), before absorption into the circulation system (Day et al., 1998; Németh et al., 2003). As an alternative, the glycosides might also be hydrolyzed after conveying inside the enterocyte via sodium‐dependent glucose transporter (SGLT 1) (Walgren et al., 2000). Thus, the produced aglycons could be passively transferred to the hepatic portal vein, or they may undergo metabolism to produce the simple forms, which are effortlessly absorbed into the hepatic portal vein through ATP‐binding cassette (ABC) transporters (Williamson et al., 2018).

### 2.4 Bioavailability of KMP

Oral delivery, the most common way of administering medication, is far higher useful, with lesser pain and risk of cross‐infection than other administrating ways (Das & Chaudhury, 2011). For flavonoids, oral delivery is also approved as the chief delivery routine. Nevertheless, most flavonoids generally show poor oral bioavailability mainly coming from their limited solubility, which rigorously diminishes their potency as the therapeutic factor. For example, the double bond between positions 2 and 3 of flavonoids is flexible to generate the planar structures, resulting in the stiff molecular organization, and then the solvent molecule is hard to enter their molecule structures (Chuang et al., 2017; Fukuhara et al., 2002). Thus, these alterations give rise to their low aqueous solubility and limited oral bioavailability. As an exemplification, the oral bioavailability of myricetin, a classic flavonol with a planar structure, in rats was estimated at approximately 9.62%, due to its low aqueous solubility of 16.60 μg/ml (Dang et al., 2014; Yao et al., 2014).

Moreover, since the extra free hydroxyl groups are effortlessly glucuronidated and sulfated in intestine cells, the gastrointestinal absorbency is detrimentally enhanced with some hydroxyl groups in a sub‐group of flavonoids, which usually leads to their poor oral absorption (Fang et al., 2017; Otake et al., 2002; Tian et al., 2009). For example, the oral bioavailability of catechin, a flavonoid mostly present in green tea, chocolate, and grapes, in rats was just 5%, which was attributed to its limited permeability owing to the 5 hydroxyl groups in its structure (Ezzat et al., 2019). Thus, to enhance the oral bioavailability of these flavonoids, increasing their saturation solubility and/or membrane permeability is needed. Besides, because of the hydroxyl and ketone groups and unsaturated double bonds, flavonoids are also susceptible to the various physical and physiological environment effectors, which possibly results in corruption or bioprocessing during depot and systemic circulation, and therefore severely restricts their effectiveness through oral absorption (Qiao et al., 2014; Xiang et al., 2017). Concerning these issues, it is necessary to suggest many useful strategies for the oral administration of flavonoids to surpass these hurdles to entirely utilize their therapeutic impacts.

In the past few decades, a growing number of approaches, such as salt formation, cocrystal formation, (Kawabata et al., 2011), prodrug approaches (Stella & Nti‐Addae, 2007), particle size reduction, complexation/solubilization, drug dispersion in carriers (Leuner & Dressman, 2000; Wang et al., 2010) have been shown to boost the bioavailability of water‐soluble drugs (see [Zhao et al., 2019]) for an overview.

Regarding the bioavailability of KMP, Cao et al. (2010) reported that an intake of 14.97 mg KMP per day represents a fasting plasma level of 57.86 nmol/L. It was shown that after intake of cooked endive including 9 mg of KMP, the main metabolite, which is identified in urine and plasma samples, was kaempferol‐3‐glucuronide. Furthermore, the highest levels of KMP in plasma were 0.1 μM after 5.8 h. Besides, almost 1.9% of total KMP was cleared from the body 24 h following the intake of the food (Dupont et al., 2004). In another study, two individuals received 500 mg of broccoli comprising 2.5 mg of KMP per 100 g for 12 days. The urine levels of glucuronide or sulfate conjugate of aglycones were 52–78 ng/ml in these subjects (Nielsen et al., 1997). In a study, seven participants following consumption of a cooked bean (*Phaseolus vulgaris* L.) diet revealed the highest excretion in urine after 2–8 h (Bonetti et al., 2007).

## 3 ANTICANCER ROLE OF KMP

KMP has been shown to have a more powerful modulating effect on a wide spectrum of cancer cell lines, such as HT29 (colon), MCF‐7 (breast), and BE2‐C (neuroblastoma), than other potential agents, such as gallic acid (Pham et al., 2018). According to the evidence, KMP may regulate cancer development and progression via its antioxidative and anti‐inflammatory actions as it restores redox hemostasis, suppresses *the NF*‐*κB* signaling pathway, and enhances the Nrf2 pathway. Based on epidemiological surveys, the dietary consumption of KMP is linked to a lower incidence of various kinds of cancer, including skin, liver, colon, ovary, pancreas, stomach, and bladder cancer (Imran, Salehi, et al., 2019). The Summary of the therapeutic functions of KMP in CRC is listed in Table 1.

**TABLE 1:** Summary of the therapeutic functions of kaempferol in colorectal cancer

Biological functions	Cancer cell lines	Source(s) of cells/tissues	Outcome(s)	Reference(s)
Anti‐oxidative and Anti‐inflammatory	Colon Tumors	Rat	Decreased in liver TBARS	(Nirmala & Ramanathan, 2011)
			Restoration of catalase, SOD, and GPx	
		Rat	The reduction of TBARS, tissue NO, serum, and tissue β‐catenin	(Hassanein et al., 2018)
			Decreased in the expression levels of PCNA and COX‐2	
Anti‐Apoptotic	SW480	Human	Increased in the expression of DR5 and DR4	(Yoshida et al., 2008)
	HCT116	Human	Induction the release of cytochrome c from mitochondria	(Li et al., 2009)
			Stimulation of the cleavage of caspase‐3	
			Increased the phosphorylation of ATM	
	HT‐29	Human	Enhancement of mitochondrial membrane permeability, cytosolic levels of cytochrome c, DNA fragmentation, chromatin condensation, and the number of early apoptotic cells	(Lee et al., 2014)
			Increased in the expression of caspase‐9, caspase‐3, caspase‐7, BAD, FAS	
			Decreased in Akt activity, caspase‐8 and BID	
Inducer of Cell Cycle Arrest	HCT8	Human	Raising the G(0)/G(1) cell fraction	(Martineti et al., 2010)
			Elevation of MT2A and SOD2	
	HT‐29	Human	Diminish the number of viable cell	(Cho & Park, 2013)
			Repressed CDK2 and CDK4 activities in addition to the protein levels of CDK2, CDK4, cyclin E, D1, and A.	
			Reduced the expression of Cdc25C, Cdc2, and cyclin B1 proteins, along with Cdc2 activity.	
Anti‐ Chemoresistant	LS174‐R	Human	Hindered the production of ROS	(Riahi‐Chebbi et al., 2019)
			Regulated the expression of JAK/STAT3, MAPK, PI3K/AKT and NF‐κB.	
	HCT116‐ and HT29‐Ox^R^	Human	Higher activities of MAPK, PI3K‐AKT, and AP‐1	(Park et al., 2021)
	HCT8‐R	Human	Reduced glucose uptake and the production of lactic acid	(Wu et al., 2022)
			Increased the expression of miR‐326 and PKM2	
Targeting miRNAs	RKO	Human	Decreased the expression of miR31, miR92a, KRAS, and c‐MYC	(Gutierrez‐Uribe et al., 2020)
			Increased AMPK and APC	
	HCT116 and DLD1	Human	Suppressed glycolysis and tumor growth through the miR‐339‐5p‐hnRNPA1/PTBP1‐PKM2 axis	(Wu et al., 2021)

Abbreviations: AMPK, AMP‐activated protein kinase; APC, adenomatous polyposis coli; ATM, ataxia telangiectasia mutated; BAD, bcl2 associated agonist of cell death; BID, BH3 interacting‐domain death agonist; CDK, cyclin‐dependent kinase; COX‐2, cyclooxygenase‐2; DR, death receptor; FAS, Fas cell surface death receptor; GPx, glutathione peroxidase; KRAS, kirsten rat sarcoma virus; MT2A, metallothionein 2A; NO, nitric oxide; PCNA, proliferating cell nuclear antigen; PKM2, pyruvate kinase M2 isoform; ROS, reactive oxygen species; SOD, superoxide dismutase; TBARS, thiobarbituric acid reactive substances.

## 4 ANTI‐OXIDATIVE AND ANTI‐INFLAMMATORY ROLES OF KMP IN CRC

It is well‐documented that KMP is regarded as a potent anti‐inflammatory and antioxidant agent in a wide spectrum of diseases, particularly cancer (Bangar et al., 2022, Imran, Rauf, et al., 2019, Imran, Salehi, et al., 2019). In this regard, in Nirmala and Ramanathan (2011) aimed to assess whether KMP has ameliorative effects on lipid peroxidation and antioxidant status in a rat model of CCR cancer. They indicated that oral administration of KMP decreased liver thiobarbituric acid reactive substances (TBARS), a byproduct of lipid peroxidation, and restored antioxidant enzymes including catalase, SOD, and glutathione peroxidase (GPx). Therefore, their research showed that KMP may be harmlessly utilized as an anticancer agent in CCR (Nirmala & Ramanathan, 2011).

In another in vivo study conducted by Hassanein et al. (2018), the anticancer impact of sulindac (SL) along with either epigallocatechin gallate (EGCG) or KMP was investigated in a rat model of CCR cancer. Their findings revealed that the combination therapy of SL with KMP and EGCG has much greater antioxidant, anti‐inflammatory, anti‐proliferating, and apoptotic functions than SL alone. Such effective anticancer actions were carried out through the reduction of TBARS, tissue nitric oxide (NO), serum, and tissue β‐catenin, and multiplicity of aberrant crypt foci (ACF). Besides, this therapy diminishes the expression levels of proliferating cell nuclear antigen (PCNA) and cyclooxygenase‐2 (COX‐2). Hence, they suggested that the utilization of SL alongside KMP or EGCG could be more effective in the treatment of CCR cancer (Hassanein et al., 2018). The same group (Hassan et al., 2021) reported that using fluoxetine (FLX) along with KMP or EGCG boosted the antioxidant, anti‐inflammatory and anti‐proliferating actions as the anti‐apoptotic activity of KMP was much greater than other agents (Hassan et al., 2021).

## 5 KMP INDUCES THE APOPTOSIS OF CRC CELLS

If a cell's DNA is damaged, the activation of Ataxia telangiectasia mutated (ATM) and ataxia telangiectasia and Rad3‐related protein (ATR) is enhanced following binding double‐strand breaks (DSBs) to the site of damage. Subsequently, ATM and ATR, as kinases, phosphorylate and activate P53, which successively suppresses the cell cycle by upregulating P21 (Abbas & Dutta, 2009; Maréchal & Zou, 2013). Apoptosis, the process of programmed cell death, will occur if the inhibition of the cell cycle by the abovementioned factors cannot tackle DNA damage before cell division. Indeed, apoptosis, the most significant obstacle to carcinogenesis, is mostly mediated by P53, which could raise the expression of proapoptotic factors including BCL2 associated x, apoptosis regulator (BAX), and BCL‐2 antagonist killer (BAK) (Amaral et al., 2010), both of which are responsible for the release of cytochrome C, activation of caspase cascade, and eventually apoptotic remains. Since the primary aim of chemotherapy medications is the stimulation of apoptosis, the major issue in almost all cancers, particularly CRC is the resilience to apoptosis because of p53 mutations.

Tumor necrosis factor‐related apoptosis‐inducing ligand (TRAIL), a potential anticancer factor, provokes apoptosis in different kinds of cancer cells, while it does not have such an effect in normal cells (Ashkenazi et al., 1999; Walczak et al., 1999). This agent engages particular pro‐apoptotic receptors, such as death receptor 5 (DR5 or TRAIL‐R2) and (DR4 or TRAIL‐R1) (Leblanc & Ashkenazi, 2003; Pan et al., 1997; Sheridan et al., 1997) as the formation of TRAIL‐DR5 complex triggers the activation of caspase‐8 and caspase‐10 (Leblanc & Ashkenazi, 2003), and ultimately caspase‐3. Due to some reports regarding the resistance of some tumor types to TRAIL, it is urgent to elaborate useful strategies to surpass this problem. In this regard, Yoshida et al. (2008) reported that the induction of apoptosis is much higher in CRC cells following combined treatment of KMP and TRAIL than single treatment with TRAIL. Furthermore, KMP noticeably increased the expression of DR5 and DR4; however, only the silencing of DR5 via siRNA effectively hinders the induction of apoptosis after the combination therapy with KMP and TRAIL, demonstrating that KMP help to improve the functions of TRAIL possibly through the upregulation of DR5. Of note, this combination therapy did not show the stimulation of apoptosis in normal cells, particularly in normal human peripheral blood mononuclear cells. Therefore, they suggested that KMP could be helpful for TRAIL‐based therapies for CRC (Yoshida et al., 2008).

Regarding the potential effects of KMP on apoptosis, Li et al. (2009) conducted a study aiming to evaluate the anti‐proliferation functions of KMP in the human HCT116 colon cancer cell line. Their findings showed that KMP stimulates p53‐dependent inhibition of cell growth and apoptosis. Correspondingly, KMP provoked the release of cytochrome c from mitochondria, the cleavage of caspase‐3, and the phosphorylation of ATM in HCT116 cells, indicating the potential of KMP as a candidate for CRC treatment and management (Li et al., 2009).

In another study conducted by Lee et al. (2014), the possible mechanism underlying the anti‐apoptotic effects of KMP was reported in the human colorectal adenocarcinoma cell line (HT‐29). They found that KMP enhances mitochondrial membrane permeability, cytosolic levels of cytochrome c, DNA fragmentation, chromatin condensation, and the number of early apoptotic cells. Besides, it increased the expression of caspase‐9, caspase‐3, caspase‐7, bcl2 associated agonist of cell death (BAD), Fas cell surface death receptor (FAS) ligand, while decreased Akt activity, uncleaved caspase‐8 and BH3 interacting‐domain death agonist (BID). Based on these results, they concluded that KMP stimulates the apoptosis of HT‐29 cells through the mitochondrial pathway and the activation of cell surface death receptors (Lee et al., 2014).

## 6 KMP INDUCES CELL CYCLE ARREST IN CRC

It has been reported that KMP stimulates differentiation of CRC cells (KNC cells), which are evaluated as alterations in cell morphology and alkaline phosphatase activity and low expression levels of connexin43 (Nakamura et al., 2005). The treatment of KNC cells with KMP enhanced the differentiation of these cells which is associated with the recovery of gap junctional intercellular communication (GJIC), elevated levels of connexin43 protein expression, and phosphorylation. On the other hand, KMP reduces the activation of Stat3 and Erk as the inhibition of Stat3 phosphorylation also provokes changes in the morphology of KNC cells similar to the abovementioned alterations in KMP‐treated cells, indicating that KMP‐stimulated differentiation could be related to the suppression of Stat3 phosphorylation. It should be noted that such effects were not detected in HCT116 cells, a poorly differentiated CRC cell line with the lowest expression of connexin43. Therefore, KMP may exert its anticancer roles through regenerating GJIC via increasing the expression and activation of connexin43 in a CRC cell line already expressing connexin43. Conversely, such an impact has not been reported for CRC cell lines with deficient connexin43 and GJIC (Nakamura et al., 2005).

In 2010, Martineti et al. (2010) reported that KMP triglyceride, a glycosylated flavonol derived from Dianthus caryophyllus cultivar, induces cell cycle arrest in HCT8 and estrogen receptor beta overexpressing CRC cells through raising the G(0)/G(1) cell fraction and antioxidant enzymes, in particular superoxide dismutase 2 (SOD2), and metallothionein 2A (MT2A), respectively (Martineti et al., 2010).

In another interesting study, Cho and Park (2013) examined the mechanism of action of KMP in suppressing the expansion of HT‐29 human colon cancer cells. Their findings revealed that KMP diminishes the number of viable cells in HT‐29 cells in a dose‐dependent fashion. Furthermore, it stimulated cell cycle arrest in G1 and G2/M phases during 6 and 12 h, respectively. Regarding the molecular mechanisms of KMP‐induced cell cycle arrest in HT‐29 cells, this flavonol represses cyclin‐dependent kinase 2 (CDK2) and CDK4 activities in addition to the protein levels of CDK2, CDK4, cyclin E, D1, and A. Moreover, it reduced the expression of Cdc25C, Cdc2, and cyclin B1 proteins, along with Cdc2 activity. Therefore, the study showed that KMP provokes cell cycle arrest in G1 and G2/M phases by suppressing the activity of CDK2, CDK4, and Cdc2. The stimulation of cell cycle arrest could be one of the potential modes of action for the anticancer impacts of KMP in CRC cells (Cho & Park, 2013). Budisan et al. (2019) were another research group, aiming to investigate the in vitro impacts of KMP in two CRC cell lines, RKO and HCT‐116. According to their results, KMP suppressed cell growth, motility, and invasion, and induced apoptosis and autophagy (Budisan et al., 2019).

## 7 KMP AS A THERAPEUTIC AGENT TO OVERCOME CHEMORESISTANCE IN CRC

Despite remarkable progress in the treatment of CRC, chemotherapy drugs, in particular 5‐fluorouracil (5‐FU) and Oxaliplatin, are the most widely utilized drugs for the treatment of CRC. As a result of the latest research, scholars have extensively tried to prevent CRC by dealing with the chemoresistance and raising the possibility of successful treatment, particularly by determining the probable mechanisms of chemoresistance, seeking solutions to mitigate them, and thereby improving the sensitivity of CRC cells to chemotherapy (Vaghari‐Tabari et al., 2020).

It is well‐known that resistance to 5‐FU chemotherapy is the main reason for failing in the treatment of CRC. Emerging combination therapy represents a successful approach to repressing cancer cells and inhibits the appearance of drug resistance. In this regard, Riahi‐Chebbi et al. (2019) evaluated the anticancer properties of some polyphenols, particularly KMP, alone or along with 5‐FU, on the human 5‐FU‐resistant CRC cells (LS174‐R). Their findings indicated that KMP can overturn 5‐FU‐resistance of LS174‐R cells through induction of apoptosis and cell cycle arrest. Additionally, this agent hindered the production of ROS and regulated the expression of some effectors, including JAK/STAT3, MAPK, PI3K/AKT, and NF‐κB. Generally, their data suggested that KMP could be regarded as a possible chemotherapeutic factor to be utilized solely or in conjunction with 5‐FU to defeat CRC drug resistance (Riahi‐Chebbi et al., 2019).

In another study designed by Park et al. (2021), the possible targeting of ribosomal S6 kinases (RSKs) by KMP was investigated in oxaliplatin (Ox)‐resistant HCT116 (HCT116‐Ox^R^) cells in comparison with Ox‐sensitive HCT116 (HCT116‐Ox^S^) cells. They reported that the activities of MAPK and PI3K‐AKT signaling pathways are considerably greater in HCT116‐Ox^R^ cells than in HCT116‐Ox^S^ cells. Furthermore, they tested various inhibitors, such as SP600125 (JNK inhibitor), SB206580 (p38 kinase inhibitor), or MK‐2206 (AKT inhibitor) on cell growth, all of which showed the inhibitory effects in the studied resistant cells. It should be noted that both studied resistant cells (HCT116‐ and HT29‐Ox^R^) cells had greater sensitivities to the suppression of cell proliferation than normal cells, leading to cell cycle arrest at the G_2_/M‐phases. Eventually, the activity of AP‐1 was shown to be reduced by KMP in resistant cells when compared with its activity in normal cells. They concluded that KMP‐mediated suppression of activator protein 1 (AP‐1) may be a significant mechanism to overcome the chemoresistance of Ox‐resistant CRC cells (Park et al., 2021).

Wu et al. (2022) have recently published an interesting paper, dealing with the therapeutic role of KMP alone or in conjunction with 5‐Fu to defeat the drug resistance of HCT8‐R cells. It is notorious that aerobic glycolysis is highly associated with tumor proliferation and chemotherapy resistance. The treatment of CRC cells with KMP substantially reduced glucose uptake and the production of lactic acid in drug‐resistant CRC cells. In relation to the possible underlying mechanism of this observation, KMP increased the expression of miR‐326, which can prevent the glycolysis process via targeting pyruvate kinase M2 isoform (PKM2) or PKM mRNA, thereby overcoming the resistance of CRC cells to 5‐Fu. Collectively, the authors of the paper implied that KMP could play a prominent role in defeating resistance to 5‐Fu therapy through modulating the miR‐326‐hnRNPA1/A2/PTBP1‐PKM2 axis (Wu et al., 2022).

## 8 TARGETING miRNAs BY KMP IN CRC

The utilization of miRNAs is one of the most reasonable explanations for conquering CRC chemoresistance. These non‐coding RNAs could be regarded as therapeutic options, molecular targets, and prognostic indicators of chemotherapy. miRNAs might soon become part of the novel clinical choices alongside the classic chemotherapeutic agents to defeat CRC chemoresistance issues. There is a broad spectrum of miRNAs, involved in reversing the chemoresistance of CRC as an in‐depth investigation of them is beyond the scope of the current paper (see [Vaghari‐Tabari et al., 2020] for an overview).

Based on our knowledge, Gutierrez‐Uribe et al. (2020) were the first to demonstrate the prohibitory impact of KMP‐3‐*O*‐glycoside on the expression levels of miR31 and miR92a, as oncological biomarkers, in a CRC cell line (RKO). Following the treatment of CRC cell line with KMP‐3‐*O*‐glycoside, the expression of miR31, miR92a, and the oncogenes, such as kirsten rat sarcoma virus (KRAS), and c‐MYC were down‐regulated, while the tumor suppressor genes, such as AMP‐activated protein kinase (AMPK) and adenomatous polyposis coli (APC) were up‐regulated. Therefore, they concluded that KMP‐3‐*O*‐glycoside could have fascinating dietary and therapeutic approaches to fighting CRC (Gutierrez‐Uribe et al., 2020).

In another related paper, Wu et al. (2021) reported that KMP diminishes the consumption of glucose, the main energy source required for the proliferation of cancer cells, eventually resulting in a decrease in the accumulation of lactic acid and the production of ATP. In terms of mechanism, KMP boosted the expression of miR‐339‐5p whose targets are heterogeneous nuclear ribonucleoprotein A1 (hnRNP A1) and polypyrimidine tract‐binding protein (PTBP1). miR‐339‐5p lessened the expression of M2‐type pyruvate kinase (PKM2) while stimulating PKM1 via directly targeting the hnRNPA1 and PTBP1. According to these findings, they suggested that KMP suppresses glycolysis and tumor growth in CRC probably through the miR‐339‐5p‐hnRNPA1/PTBP1‐PKM2 axis, indicating a novel elucidation for the molecular aspects of the anticancer roles of KMP (Wu et al., 2021).

## 9 CONCLUSIONS

As mentioned above, KMP has a wide range of therapeutic functions, including anti‐apoptotic, anti‐inflammatory, anti‐oxidative, anti‐proliferative, and anti‐chemoresistant in CRC (Table 1). However, the problem is that almost all of the studies conducted to assess the therapeutic actions of KMP in the treatment of CRC, are in vitro studies and only two in vivo studies have been published. Therefore, there is an urgent need to comprehensively investigate its curative effects in different animal models of CRC to assess its long‐term impacts as a safe therapeutic agent. Correspondingly, it is possible that using kaempferol‐based nanoparticles will give good grounds for expecting that these types of technologies may boost its bioavailability and efficiency in the treatment of cancer, in particular CRC. In addition to more clinical studies which should be carried out for unraveling the therapeutic potentials of KMP in CRC, using its various glycosylated forms could also be immensely helpful. Of note, the combination therapy of KMP with other anticancer agents, such as SL and EGCG has been shown to improve their effectiveness in CRC therapy. Overall, a good deal of preclinical studies has approved the function of KMP in the treatment of CRC. However, there are numerous doubts regarding the therapeutic potency of KMP in CRC therapy which should be addressed by elite scientists all over the world

## FUNDING INFORMATION

This research did not receive any specific grant from funding agencies in the public, commercial, or not‐for‐profit sectors.

## CONFLICTS OF INTEREST

The authors report there are no competing interests to declare.

## ETHICS APPROVAL

Not applicable.

## PATIENT CONSENT STATEMENT

Not applicable.

## PERMISSION TO REPRODUCE MATERIAL FROM OTHER SOURCES

Not applicable.

## CLINICAL TRIAL REGISTRATION

Not applicable.

## References

[phy215488-bib-0001] Abbas, T. , & Dutta, A. (2009). p21 in cancer: Intricate networks and multiple activities. Nature Reviews Cancer, 9, 400–414. 19440234 10.1038/nrc2657 PMC2722839

[phy215488-bib-0002] Akiyama, Y. , Kimura, Y. , Enatsu, R. , Mikami, T. , Wanibuchi, M. , & Mikuni, N. (2018). Advantages and disadvantages of combined chemotherapy with Carmustine wafer and bevacizumab in patients with newly diagnosed glioblastoma: A single‐institutional experience. World Neurosurgery, 113, e508–e514. 29476996 10.1016/j.wneu.2018.02.070

[phy215488-bib-0003] Alam, W. , Khan, H. , Shah, M. A. , Cauli, O. , & Saso, L. (2020). Kaempferol as a dietary anti‐inflammatory agent: Current therapeutic standing. Molecules, 25(18), 4073. 10.3390/molecules25184073 PMC7570692 32906577

[phy215488-bib-0004] Alvarez, A. I. , Real, R. , Pérez, M. , Mendoza, G. , Prieto, J. G. , & Merino, G. (2010). Modulation of the activity of ABC transporters (P‐glycoprotein, MRP2, BCRP) by flavonoids and drug response. Journal of Pharmaceutical Sciences, 99, 598–617. 19544374 10.1002/jps.21851

[phy215488-bib-0005] Amaral, J. D. , Xavier, J. M. , Steer, C. J. , & Rodrigues, C. M. (2010). The role of p53 in apoptosis. Discovery Medicine, 9, 145–152. 20193641

[phy215488-bib-0006] Aquina, C. T. , Mohile, S. G. , Tejani, M. A. , Becerra, A. Z. , Xu, Z. , Hensley, B. J. , Arsalani‐Zadeh, R. , Boscoe, F. P. , Schymura, M. J. , Noyes, K. , Monson, J. R. , & Fleming, F. J. (2017). The impact of age on complications, survival, and cause of death following colon cancer surgery. British Journal of Cancer, 116, 389–397. 28056465 10.1038/bjc.2016.421 PMC5294480

[phy215488-bib-0007] Ashkenazi, A. , Pai, R. C. , Fong, S. , Leung, S. , Lawrence, D. A. , Marsters, S. A. , Blackie, C. , Chang, L. , Mcmurtrey, A. E. , Hebert, A. , Deforge, L. , Koumenis, I. L. , Lewis, D. , Harris, L. , Bussiere, J. , Koeppen, H. , Shahrokh, Z. , & Schwall, R. H. (1999). Safety and antitumor activity of recombinant soluble Apo2 ligand. The Journal of Clinical Investigation, 104, 155–162. 10411544 10.1172/JCI6926 PMC408479

[phy215488-bib-0008] Bangar, S. P. , Chaudhary, V. , Sharma, N. , Bansal, V. , Ozogul, F. , & Lorenzo, J. M. (2022). Kaempferol: A flavonoid with wider biological activities and its applications. Critical Reviews in Food Science and Nutrition, 1–25. 10.1080/10408398.2022.2067121 35468008

[phy215488-bib-0009] Bar‐Shalom, R. , Bergman, M. , Grossman, S. , Azzam, N. , Sharvit, L. , & Fares, F. (2019). Inula Viscosa extract inhibits growth of colorectal cancer cells in vitro and in vivo through induction of apoptosis. Frontiers in Oncology, 9, 227. 31024836 10.3389/fonc.2019.00227 PMC6469364

[phy215488-bib-0010] Bonetti, A. , Marotti, I. , & Dinelli, G. (2007). Urinary excretion of kaempferol from common beans (Phaseolus vulgaris L.) in humans. International Journal of Food Sciences and Nutrition, 58, 261–269. 17566888 10.1080/09637480601154228

[phy215488-bib-0011] Bray, F. , Ferlay, J. , Soerjomataram, I. , Siegel, R. L. , Torre, L. A. , & Jemal, A. (2018). Global cancer statistics 2018: GLOBOCAN estimates of incidence and mortality worldwide for 36 cancers in 185 countries. CA: A Cancer Journal for Clinicians, 68, 394–424. 30207593 10.3322/caac.21492

[phy215488-bib-0012] Budisan, L. , Gulei, D. , Jurj, A. , Braicu, C. , Zanoaga, O. , Cojocneanu, R. , Pop, L. , Raduly, L. , Barbat, A. , Moldovan, A. , Moldovan, C. , Tigu, A. B. , Ionescu, C. , Atanasov, A. G. , Irimie, A. , & Berindan‐Neagoe, I. (2019). Inhibitory effect of CAPE and kaempferol in colon cancer cell lines‐possible implications in new therapeutic strategies. International Journal of Molecular Sciences, 20, 1199. 10.3390/ijms20051199 PMC6429399 30857282

[phy215488-bib-0013] Cao, J. , Zhang, Y. , Chen, W. , & Zhao, X. (2010). The relationship between fasting plasma concentrations of selected flavonoids and their ordinary dietary intake. The British Journal of Nutrition, 103, 249–255. 19747418 10.1017/S000711450999170X

[phy215488-bib-0014] Chen, W. , Xiao, Z. , Wang, Y. , Wang, J. , Zhai, R. , Lin‐Wang, K. , Espley, R. , Ma, F. , & Li, P. (2021). Competition between anthocyanin and kaempferol glycosides biosynthesis affects pollen tube growth and seed set of malus. Horticulture Research, 8, 173. 34333541 10.1038/s41438-021-00609-9 PMC8325685

[phy215488-bib-0015] Cho, H. J. , & Park, J. H. (2013). Kaempferol induces cell cycle arrest in HT‐29 human colon cancer cells. Journal of cancer prevention, 18, 257–263. 25337553 10.15430/JCP.2013.18.3.257 PMC4189462

[phy215488-bib-0016] Chuang, S. Y. , Lin, Y. K. , Lin, C. F. , Wang, P. W. , Chen, E. L. , & Fang, J. Y. (2017). Elucidating the skin delivery of aglycone and glycoside flavonoids: How the structures affect cutaneous absorption. Nutrients, 9, 1304. 10.3390/nu9121304 PMC5748754 29189718

[phy215488-bib-0017] Crespy, V. , Morand, C. , Besson, C. , Cotelle, N. , Vézin, H. , Demigné, C. , & Rémésy, C. (2003). The splanchnic metabolism of flavonoids highly differed according to the nature of the compound. American Journal of Physiology. Gastrointestinal and Liver Physiology, 284, G980–G988. 12736148 10.1152/ajpgi.00223.2002

[phy215488-bib-0018] Dai, J. , & Mumper, R. J. (2010). Plant phenolics: Extraction, analysis and their antioxidant and anticancer properties. Molecules, 15, 7313–7352. 20966876 10.3390/molecules15107313 PMC6259146

[phy215488-bib-0019] Dang, Y. , Lin, G. , Xie, Y. , Duan, J. , Ma, P. , Li, G. , & Ji, G. (2014). Quantitative determination of myricetin in rat plasma by ultra performance liquid chromatography tandem mass spectrometry and its absolute bioavailability. Drug Research (Stuttg), 64, 516–522. 10.1055/s-0033-1363220 24357136

[phy215488-bib-0020] D'archivio, M. , Filesi, C. , Di Benedetto, R. , Gargiulo, R. , Giovannini, C. , & Masella, R. (2007). Polyphenols, dietary sources and bioavailability. Annali dell'Istituto Superiore di Sanità, 43, 348–361. 18209268

[phy215488-bib-0021] Das, S. , & Chaudhury, A. (2011). Recent advances in lipid nanoparticle formulations with solid matrix for oral drug delivery. AAPS PharmSciTech, 12, 62–76. 21174180 10.1208/s12249-010-9563-0 PMC3066374

[phy215488-bib-0022] Day, A. J. , Dupont, M. S. , Ridley, S. , Rhodes, M. , Rhodes, M. J. , Morgan, M. R. , & Williamson, G. (1998). Deglycosylation of flavonoid and isoflavonoid glycosides by human small intestine and liver beta‐glucosidase activity. FEBS Letters, 436, 71–75. 9771896 10.1016/s0014-5793(98)01101-6

[phy215488-bib-0023] Duan, L. , Ding, W. , Liu, X. , Cheng, X. , Cai, J. , Hua, E. , & Jiang, H. (2017). Biosynthesis and engineering of kaempferol in Saccharomyces cerevisiae. Microbial Cell Factories, 16, 165. 28950867 10.1186/s12934-017-0774-x PMC5615808

[phy215488-bib-0024] Dupont, M. S. , Day, A. J. , Bennett, R. N. , Mellon, F. A. , & Kroon, P. A. (2004). Absorption of kaempferol from endive, a source of kaempferol‐3‐glucuronide, in humans. European Journal of Clinical Nutrition, 58, 947–954. 15164116 10.1038/sj.ejcn.1601916

[phy215488-bib-0025] Ezzat, H. M. , Elnaggar, Y. S. R. , & Abdallah, O. Y. (2019). Improved oral bioavailability of the anticancer drug catechin using chitosomes: Design, in‐vitro appraisal and in‐vivo studies. International Journal of Pharmaceutics, 565, 488–498. 31100382 10.1016/j.ijpharm.2019.05.034

[phy215488-bib-0026] Fang, Y. , Cao, W. , Xia, M. , Pan, S. , & Xu, X. (2017). Study of structure and permeability relationship of flavonoids in Caco‐2 cells. Nutrients, 9, 1301. 10.3390/nu9121301 PMC5748751 29186068

[phy215488-bib-0027] Fukuhara, K. , Nakanishi, I. , Kansui, H. , Sugiyama, E. , Kimura, M. , Shimada, T. , Urano, S. , Yamaguchi, K. , & Miyata, N. (2002). Enhanced radical‐scavenging activity of a planar catechin analogue. Journal of the American Chemical Society, 124, 5952–5953. 12022823 10.1021/ja0178259

[phy215488-bib-0028] Gamage, S. M. K. , Nanayakkara, S. , Macfarlane, L. , Hewage, D. , Cheng, T. , Aktar, S. , Lu, C. T. , Dissabandara, L. , Islam, F. , Lam, A. K. , & Gopalan, V. (2022). Heme oxygenase‐1 & 2 and their potential contribution in heme induced colorectal carcinogenesis. Pathology, Research and Practice, 233, 153885. 35428017 10.1016/j.prp.2022.153885

[phy215488-bib-0029] Ghosh, D. , Dey, S. K. , & Saha, C. (2014). Antagonistic effects of black tea against gamma radiation‐induced oxidative damage to normal lymphocytes in comparison with cancerous K562 cells. Radiation and Environmental Biophysics, 53, 695–704. 24981250 10.1007/s00411-014-0551-8

[phy215488-bib-0030] Giordano, A. , & Tommonaro, G. (2019). Curcumin and Cancer. Nutrients, 11, 2376. 10.3390/nu11102376 PMC6835707 31590362

[phy215488-bib-0031] Grivennikov, S. I. , & Cominelli, F. (2016). Colitis‐associated and sporadic colon cancers: Different diseases, different mutations? Gastroenterology, 150, 808–810. 26924087 10.1053/j.gastro.2016.02.062

[phy215488-bib-0032] Gutierrez‐Uribe, J. A. , Salinas‐Santander, M. , Serna‐Guerrero, D. , Serna‐Saldivar, S. R. O. , Rivas‐Estilla, A. M. , & Rios‐Ibarra, C. P. (2020). Inhibition of miR31 and miR92a as oncological biomarkers in RKO colon cancer cells treated with Kaempferol‐3‐O‐glycoside isolated from black bean. Journal of Medicinal Food, 23, 50–55. 31441682 10.1089/jmf.2019.0059

[phy215488-bib-0033] Hassan, E. S. G. , Hassanein, N. M. , & Sayed Ahmed, H. M. (2021). Probing the chemoprevention potential of the antidepressant fluoxetine combined with epigallocatechin gallate or kaempferol in rats with induced early stage colon carcinogenesis. Journal of Pharmacological Sciences, 145, 29–41. 33357777 10.1016/j.jphs.2020.10.005

[phy215488-bib-0034] Hassanein, N. M. A. , Hassan, E. S. G. , Hegab, A. M. , & Elahl, H. M. S. (2018). Chemopreventive effect of sulindac in combination with epigallocatechin gallate or kaempferol against 1,2‐dimethyl hydrazine‐induced preneoplastic lesions in rats: A comparative study. Journal of Biochemical and Molecular Toxicology, 32, e22198. 29999212 10.1002/jbt.22198

[phy215488-bib-0035] Heijnen, C. G. , Haenen, G. R. , Van Acker, F. A. , Van Der Vijgh, W. J. , & Bast, A. (2001). Flavonoids as peroxynitrite scavengers: The role of the hydroxyl groups. Toxicology In Vitro, 15, 3–6. 11259863 10.1016/s0887-2333(00)00053-9

[phy215488-bib-0036] Huang, W. W. , Tsai, S. C. , Peng, S. F. , Lin, M. W. , Chiang, J. H. , Chiu, Y. J. , Fushiya, S. , Tseng, M. T. , & Yang, J. S. (2013). Kaempferol induces autophagy through AMPK and AKT signaling molecules and causes G2/M arrest via downregulation of CDK1/cyclin B in SK‐HEP‐1 human hepatic cancer cells. International Journal of Oncology, 42, 2069–2077. 23591552 10.3892/ijo.2013.1909

[phy215488-bib-0037] Imran, M. , Rauf, A. , Shah, Z. A. , Saeed, F. , Imran, A. , Arshad, M. U. , Ahmad, B. , Bawazeer, S. , Atif, M. , Peters, D. G. , & Mubarak, M. S. (2019). Chemo‐preventive and therapeutic effect of the dietary flavonoid kaempferol: A comprehensive review. Phytotherapy Research, 33, 263–275. 30402931 10.1002/ptr.6227

[phy215488-bib-0038] Imran, M. , Salehi, B. , Sharifi‐Rad, J. , Aslam Gondal, T. , Saeed, F. , Imran, A. , Shahbaz, M. , Tsouh Fokou, P. V. , Umair Arshad, M. , Khan, H. , Guerreiro, S. G. , Martins, N. , & Estevinho, L. M. (2019). Kaempferol: A key emphasis to its anticancer potential. Molecules, 24, 2277. 10.3390/molecules24122277 PMC6631472 31248102

[phy215488-bib-0039] Issa, I. A. , & Noureddine, M. (2017). Colorectal cancer screening: An updated review of the available options. World Journal of Gastroenterology, 23, 5086–5096. 28811705 10.3748/wjg.v23.i28.5086 PMC5537177

[phy215488-bib-0040] Jiang, H. , Engelhardt, U. H. , Thräne, C. , Maiwald, B. , & Stark, J. (2015). Determination of flavonol glycosides in green tea, oolong tea and black tea by UHPLC compared to HPLC. Food Chemistry, 183, 30–35. 25863606 10.1016/j.foodchem.2015.03.024

[phy215488-bib-0041] Jones, J. A. , Vernacchio, V. R. , Sinkoe, A. L. , Collins, S. M. , Ibrahim, M. H. A. , Lachance, D. M. , Hahn, J. , & Koffas, M. A. G. (2016). Experimental and computational optimization of an Escherichia coli co‐culture for the efficient production of flavonoids. Metabolic Engineering, 35, 55–63. 26860871 10.1016/j.ymben.2016.01.006

[phy215488-bib-0042] Kang, J. W. , Kim, J. H. , Song, K. , Kim, S. H. , Yoon, J. H. , & Kim, K. S. (2010). Kaempferol and quercetin, components of Ginkgo biloba extract (EGb 761), induce caspase‐3‐dependent apoptosis in oral cavity cancer cells. Phytotherapy Research, 24(Suppl 1), S77–S82. 19585476 10.1002/ptr.2913

[phy215488-bib-0043] Kawabata, Y. , Wada, K. , Nakatani, M. , Yamada, S. , & Onoue, S. (2011). Formulation design for poorly water‐soluble drugs based on biopharmaceutics classification system: Basic approaches and practical applications. International Journal of Pharmaceutics, 420, 1–10. 21884771 10.1016/j.ijpharm.2011.08.032

[phy215488-bib-0044] Klaunig, J. E. , & Kamendulis, L. M. (2004). The role of oxidative stress in carcinogenesis. Annual Review of Pharmacology and Toxicology, 44, 239–267. 10.1146/annurev.pharmtox.44.101802.121851 14744246

[phy215488-bib-0045] Leblanc, H. N. , & Ashkenazi, A. (2003). Apo2L/TRAIL and its death and decoy receptors. Cell Death and Differentiation, 10, 66–75. 12655296 10.1038/sj.cdd.4401187

[phy215488-bib-0046] Lee, H. S. , Cho, H. J. , Yu, R. , Lee, K. W. , Chun, H. S. , & Park, J. H. (2014). Mechanisms underlying apoptosis‐inducing effects of kaempferol in HT‐29 human colon cancer cells. International Journal of Molecular Sciences, 15, 2722–2737. 24549175 10.3390/ijms15022722 PMC3958878

[phy215488-bib-0047] Lehtonen, H. M. , Lehtinen, O. , Suomela, J. P. , Viitanen, M. , & Kallio, H. (2010). Flavonol glycosides of sea buckthorn (Hippophaë rhamnoides ssp. sinensis) and lingonberry (Vaccinium vitis‐idaea) are bioavailable in humans and monoglucuronidated for excretion. Journal of Agricultural and Food Chemistry, 58, 620–627. 20050706 10.1021/jf9029942

[phy215488-bib-0048] Leuner, C. , & Dressman, J. (2000). Improving drug solubility for oral delivery using solid dispersions. European Journal of Pharmaceutics and Biopharmaceutics, 50, 47–60. 10840192 10.1016/s0939-6411(00)00076-x

[phy215488-bib-0049] Li, H. , Zhang, C. , Zhang, H. , & Li, H. (2021). Xanthine oxidoreductase promotes the progression of colitis‐associated colorectal cancer by causing DNA damage and mediating macrophage M1 polarization. European Journal of Pharmacology, 906, 174270. 34171392 10.1016/j.ejphar.2021.174270

[phy215488-bib-0050] Li, W. , Du, B. , Wang, T. , Wang, S. , & Zhang, J. (2009). Kaempferol induces apoptosis in human HCT116 colon cancer cells via the ataxia‐telangiectasia mutated‐p53 pathway with the involvement of p53 upregulated modulator of apoptosis. Chemico‐Biological Interactions, 177, 121–127. 19028473 10.1016/j.cbi.2008.10.048

[phy215488-bib-0051] Madni, A. , Batool, A. , Noreen, S. , Maqbool, I. , Rehman, F. , Kashif, P. M. , Tahir, N. , & Raza, A. (2017). Novel nanoparticulate systems for lung cancer therapy: An updated review. Journal of Drug Targeting, 25, 499–512. 28151021 10.1080/1061186X.2017.1289540

[phy215488-bib-0052] Majeed, I. , Rizwan, K. , Ashar, A. , Rasheed, T. , Amarowicz, R. , Kausar, H. , Zia‐Ul‐Haq, M. , & Marceanu, L. G. (2021). A comprehensive review of the Ethnotraditional uses and biological and pharmacological potential of the genus mimosa. International Journal of Molecular Sciences, 22, 7463. 34299082 10.3390/ijms22147463 PMC8307580

[phy215488-bib-0053] Malla, S. , Pandey, R. P. , Kim, B. G. , & Sohng, J. K. (2013). Regiospecific modifications of naringenin for astragalin production in *Escherichia coli* . Biotechnology and Bioengineering, 110, 2525–2535. 23568509 10.1002/bit.24919

[phy215488-bib-0054] Maréchal, A. , & Zou, L. (2013). DNA damage sensing by the ATM and ATR kinases. Cold Spring Harbor Perspectives in Biology, 5, a012716. 24003211 10.1101/cshperspect.a012716 PMC3753707

[phy215488-bib-0055] Martineti, V. , Tognarini, I. , Azzari, C. , Carbonell Sala, S. , Clematis, F. , Dolci, M. , Lanzotti, V. , Tonelli, F. , Brandi, M. L. , & Curir, P. (2010). Inhibition of in vitro growth and arrest in the G0/G1 phase of HCT8 line human colon cancer cells by kaempferide triglycoside from Dianthus caryophyllus. Phytotherapy Research, 24, 1302–1308. 20104502 10.1002/ptr.3105

[phy215488-bib-0056] Mollaei, H. , Safaralizadeh, R. , & Rostami, Z. (2019). MicroRNA replacement therapy in cancer. Journal of Cellular Physiology, 234, 12369–12384. 30605237 10.1002/jcp.28058 PMC13484118

[phy215488-bib-0057] Nakamura, Y. , Chang, C. C. , Mori, T. , Sato, K. , Ohtsuki, K. , Upham, B. L. , & Trosko, J. E. (2005). Augmentation of differentiation and gap junction function by kaempferol in partially differentiated colon cancer cells. Carcinogenesis, 26, 665–671. 15618237 10.1093/carcin/bgi003

[phy215488-bib-0058] Narayan, S. , & Roy, D. (2003). Role of APC and DNA mismatch repair genes in the development of colorectal cancers. Molecular Cancer, 2, 41. 14672538 10.1186/1476-4598-2-41 PMC317355

[phy215488-bib-0059] Németh, K. , Plumb, G. W. , Berrin, J. G. , Juge, N. , Jacob, R. , Naim, H. Y. , Williamson, G. , Swallow, D. M. , & Kroon, P. A. (2003). Deglycosylation by small intestinal epithelial cell beta‐glucosidases is a critical step in the absorption and metabolism of dietary flavonoid glycosides in humans. European Journal of Nutrition, 42, 29–42. 12594539 10.1007/s00394-003-0397-3

[phy215488-bib-0060] Nielsen, S. E. , Kall, M. , Justesen, U. , Schou, A. , & Dragsted, L. O. (1997). Human absorption and excretion of flavonoids after broccoli consumption. Cancer Letters, 114, 173–174. 9103283 10.1016/s0304-3835(97)04654-5

[phy215488-bib-0061] Nirmala, P. , & Ramanathan, M. (2011). Effect of kaempferol on lipid peroxidation and antioxidant status in 1,2‐dimethyl hydrazine induced colorectal carcinoma in rats. European Journal of Pharmacology, 654, 75–79. 21172346 10.1016/j.ejphar.2010.11.034

[phy215488-bib-0062] Otake, Y. , Hsieh, F. , & Walle, T. (2002). Glucuronidation versus oxidation of the flavonoid galangin by human liver microsomes and hepatocytes. Drug Metabolism and Disposition, 30, 576–581. 11950790 10.1124/dmd.30.5.576

[phy215488-bib-0063] Pan, G. , Ni, J. , Wei, Y. F. , Yu, G. , Gentz, R. , & Dixit, V. M. (1997). An antagonist decoy receptor and a death domain‐containing receptor for TRAIL. Science, 277, 815–818. 9242610 10.1126/science.277.5327.815

[phy215488-bib-0064] Park, J. , Lee, G. E. , An, H. J. , Lee, C. J. , Cho, E. S. , Kang, H. C. , Lee, J. Y. , Lee, H. S. , Choi, J. S. , Kim, D. J. , Choi, J. S. , & Cho, Y. Y. (2021). Kaempferol sensitizes cell proliferation inhibition in oxaliplatin‐resistant colon cancer cells. Archives of Pharmacal Research, 44, 1091–1108. 34750753 10.1007/s12272-021-01358-y

[phy215488-bib-0065] Pei, J. , Dong, P. , Wu, T. , Zhao, L. , Fang, X. , Cao, F. , Tang, F. , & Yue, Y. (2016). Metabolic engineering of Escherichia coli for astragalin biosynthesis. Journal of Agricultural and Food Chemistry, 64, 7966–7972. 27730814 10.1021/acs.jafc.6b03447

[phy215488-bib-0066] Pham, H. N. T. , Sakoff, J. A. , Vuong, Q. V. , Bowyer, M. C. , & Scarlett, C. J. (2018). Comparative cytotoxic activity between kaempferol and gallic acid against various cancer cell lines. Data in Brief, 21, 1033–1036. 30450396 10.1016/j.dib.2018.10.121 PMC6226582

[phy215488-bib-0067] Qiao, L. , Sun, Y. , Chen, R. , Fu, Y. , Zhang, W. , Li, X. , Chen, J. , Shen, Y. , & Ye, X. (2014). Sonochemical effects on 14 flavonoids common in citrus: Relation to stability. PLoS One, 9, e87766. 24516562 10.1371/journal.pone.0087766 PMC3916345

[phy215488-bib-0068] Rajendran, P. , Rengarajan, T. , Nandakumar, N. , Palaniswami, R. , Nishigaki, Y. , & Nishigaki, I. (2014). Kaempferol, a potential cytostatic and cure for inflammatory disorders. European Journal of Medicinal Chemistry, 86, 103–112. 25147152 10.1016/j.ejmech.2014.08.011

[phy215488-bib-0069] Riahi‐Chebbi, I. , Souid, S. , Othman, H. , Haoues, M. , Karoui, H. , Morel, A. , Srairi‐Abid, N. , Essafi, M. , & Essafi‐Benkhadir, K. (2019). The phenolic compound kaempferol overcomes 5‐fluorouracil resistance in human resistant LS174 colon cancer cells. Scientific Reports, 9, 195. 30655588 10.1038/s41598-018-36808-z PMC6336835

[phy215488-bib-0070] Roncucci, L. , & Mariani, F. (2015). Prevention of colorectal cancer: How many tools do we have in our basket? European Journal of Internal Medicine, 26, 752–756. 26499755 10.1016/j.ejim.2015.08.019

[phy215488-bib-0071] Sheridan, J. P. , Marsters, S. A. , Pitti, R. M. , Gurney, A. , Skubatch, M. , Baldwin, D. , Ramakrishnan, L. , Gray, C. L. , Baker, K. , Wood, W. I. , Goddard, A. D. , Godowski, P. , & Ashkenazi, A. (1997). Control of TRAIL‐induced apoptosis by a family of signaling and decoy receptors. Science, 277, 818–821. 9242611 10.1126/science.277.5327.818

[phy215488-bib-0072] Stella, V. J. , & Nti‐Addae, K. W. (2007). Prodrug strategies to overcome poor water solubility. Advanced Drug Delivery Reviews, 59, 677–694. 17628203 10.1016/j.addr.2007.05.013

[phy215488-bib-0073] Tian, X. J. , Yang, X. W. , Yang, X. , & Wang, K. (2009). Studies of intestinal permeability of 36 flavonoids using Caco‐2 cell monolayer model. International Journal of Pharmaceutics, 367, 58–64. 18848870 10.1016/j.ijpharm.2008.09.023

[phy215488-bib-0074] Vaghari‐Tabari, M. , Majidinia, M. , Moein, S. , Qujeq, D. , Asemi, Z. , Alemi, F. , Mohamadzadeh, R. , Targhazeh, N. , Safa, A. , & Yousefi, B. (2020). MicroRNAs and colorectal cancer chemoresistance: New solution for old problem. Life Sciences, 259, 118255. 32818543 10.1016/j.lfs.2020.118255

[phy215488-bib-0075] Walczak, H. , Miller, R. E. , Ariail, K. , Gliniak, B. , Griffith, T. S. , Kubin, M. , Chin, W. , Jones, J. , Woodward, A. , Le, T. , Smith, C. , Smolak, P. , Goodwin, R. G. , Rauch, C. T. , Schuh, J. C. , & Lynch, D. H. (1999). Tumoricidal activity of tumor necrosis factor‐related apoptosis‐inducing ligand in vivo. Nature Medicine, 5, 157–163. 10.1038/5517 9930862

[phy215488-bib-0076] Walgren, R. A. , Lin, J. T. , Kinne, R. K. , & Walle, T. (2000). Cellular uptake of dietary flavonoid quercetin 4′‐beta‐glucoside by sodium‐dependent glucose transporter SGLT1. The Journal of Pharmacology and Experimental Therapeutics, 294, 837–843. 10945831

[phy215488-bib-0077] Wang, L. , Tu, Y. C. , Lian, T. W. , Hung, J. T. , Yen, J. H. , & Wu, M. J. (2006). Distinctive antioxidant and antiinflammatory effects of flavonols. Journal of Agricultural and Food Chemistry, 54, 9798–9804. 17177504 10.1021/jf0620719

[phy215488-bib-0078] Wang, Y. , Yan, Y. , Cui, J. , Hosta‐Rigau, L. , Heath, J. K. , Nice, E. C. , & Caruso, F. (2010). Encapsulation of water‐insoluble drugs in polymer capsules prepared using mesoporous silica templates for intracellular drug delivery. Advanced Materials, 22, 4293–4297. 20564713 10.1002/adma.201001497

[phy215488-bib-0079] Williamson, G. , Kay, C. D. , & Crozier, A. (2018). The bioavailability, transport, and bioactivity of dietary flavonoids: A review from a historical perspective. Comprehensive Reviews in Food Science and Food Safety, 17, 1054–1112. 33350159 10.1111/1541-4337.12351

[phy215488-bib-0080] Wu, H. , Cui, M. , Li, C. , Li, H. , Dai, Y. , Cui, K. , & Li, Z. (2021). Kaempferol reverses aerobic glycolysis via miR‐339‐5p‐mediated PKM alternative splicing in colon cancer cells. Journal of Agricultural and Food Chemistry, 69, 3060–3068. 33663206 10.1021/acs.jafc.0c07640

[phy215488-bib-0081] Wu, H. , Du, J. , Li, C. , Li, H. , Guo, H. , & Li, Z. (2022). Kaempferol can reverse the 5‐Fu resistance of colorectal cancer cells by inhibiting PKM2‐mediated glycolysis. International Journal of Molecular Sciences, 23, 3544. 35408903 10.3390/ijms23073544 PMC8998549

[phy215488-bib-0082] Xiang, D. , Wang, C. G. , Wang, W. Q. , Shi, C. Y. , Xiong, W. , Wang, M. D. , & Fang, J. G. (2017). Gastrointestinal stability of dihydromyricetin, myricetin, and myricitrin: An in vitro investigation. International Journal of Food Sciences and Nutrition, 68, 704–711. 28114854 10.1080/09637486.2016.1276518

[phy215488-bib-0083] Xiao, J. , Muzashvili, T. S. , & Georgiev, M. I. (2014). Advances in the biotechnological glycosylation of valuable flavonoids. Biotechnology Advances, 32, 1145–1156. 24780153 10.1016/j.biotechadv.2014.04.006

[phy215488-bib-0084] Xiong, D. , Lu, S. , Wu, J. , Liang, C. , Wang, W. , Wang, W. , Jin, J. M. , & Tang, S. Y. (2017). Improving key enzyme activity in phenylpropanoid pathway with a designed biosensor. Metabolic Engineering, 40, 115–123. 28111248 10.1016/j.ymben.2017.01.006

[phy215488-bib-0085] Yamagishi, H. , Kuroda, H. , Imai, Y. , & Hiraishi, H. (2016). Molecular pathogenesis of sporadic colorectal cancers. Chinese Journal of Cancer, 35, 4. 26738600 10.1186/s40880-015-0066-y PMC4704376

[phy215488-bib-0086] Yang, Y. (2015). Cancer immunotherapy: Harnessing the immune system to battle cancer. The Journal of Clinical Investigation, 125, 3335–3337. 26325031 10.1172/JCI83871 PMC4588312

[phy215488-bib-0087] Yao, Y. , Lin, G. , Xie, Y. , Ma, P. , Li, G. , Meng, Q. , & Wu, T. (2014). Preformulation studies of myricetin: A natural antioxidant flavonoid. Pharmazie, 69, 19–26. 24601218

[phy215488-bib-0088] Yoshida, T. , Konishi, M. , Horinaka, M. , Yasuda, T. , Goda, A. E. , Taniguchi, H. , Yano, K. , Wakada, M. , & Sakai, T. (2008). Kaempferol sensitizes colon cancer cells to TRAIL‐induced apoptosis. Biochemical and Biophysical Research Communications, 375, 129–133. 18680719 10.1016/j.bbrc.2008.07.131

[phy215488-bib-0089] Zhao, J. , Yang, J. , & Xie, Y. (2019). Improvement strategies for the oral bioavailability of poorly water‐soluble flavonoids: An overview. International Journal of Pharmaceutics, 570, 118642. 31446024 10.1016/j.ijpharm.2019.118642

